# Posttraumatic Cranial Cystic Fibrous Dysplasia

**DOI:** 10.1155/2011/680401

**Published:** 2011-08-25

**Authors:** Arata Tomiyama, Kazuya Aoki, Haruo Nakayama, Hideaki Izukura, Hitoshi Kimura, Jun-ichi Harashina, Keisuke Ito, Morito Hayashi, Norihiko Saito, Takatoshi Sakurai, Toshiaki Oharaseki, Hitoshi Terada, Satoshi Iwabuchi

**Affiliations:** ^1^Division of Metastasis and Invasion Signaling, National Cancer Center, Research Institute, Tokyo 104-0045, Japan; ^2^Department of Neurosurgery, Toho University Ohashi Medical Center, 2-17-6 Ohashi, Meguro-ku, Tokyo 153-8515, Japan; ^3^Department of Pathology, Toho University Ohashi Medical Center, Tokyo 153-8515, Japan; ^4^Department of Radiology, Toho University Sakura Medical Center, Chiba 285-8741, Japan

## Abstract

A 14-year-old was girl admitted to our hospital with a subcutaneous mass of the occipital head. The mass had grown for 6 years, after she had sustained a head injury at the age of 6, and was located directly under a previous wound. Skull X-ray Photograph (xp), computed tomography (CT), and magnetic resonance imaging (MRI) showed a bony defect and cystic changes in the skull corresponding to a subcutaneous mass. Bone scintigraphy revealed partial accumulation. The patient underwent total removal of the skull mass, and the diagnosis from the pathological findings of the cyst wall was fibrous dysplasia (FD). The radiographic findings for cystic cranial FD can be various. Progressive skull disease has been reported to be associated with head trauma, but the relationship between cranial FD and head trauma has not been previously reported. Previous studies have suggested that *c-fos* gene expression is a key mechanism in injury-induced FD.

## 1. Introduction

Fibrous dysplasia (FD) is a progressive systemic bone tumor-like lesion involving the skull that develops in young people [1]. It primarily occurs in the facial or frontal regions of the head, including the skull base, and rarely affects the occipital or convexity region [2]. The exact pathogenesis of FD is still unknown, but recently, a genetic abnormality has been reported as a cause of FD [3]. In this paper, we describe what we believe to be the first case of cranial FD possibly triggered by head injury that presented as a growing mass in the occipital region, which was difficult to diagnose based on clinical course and radiographic images.

## 2. Case Presentation

**Figure 1:**
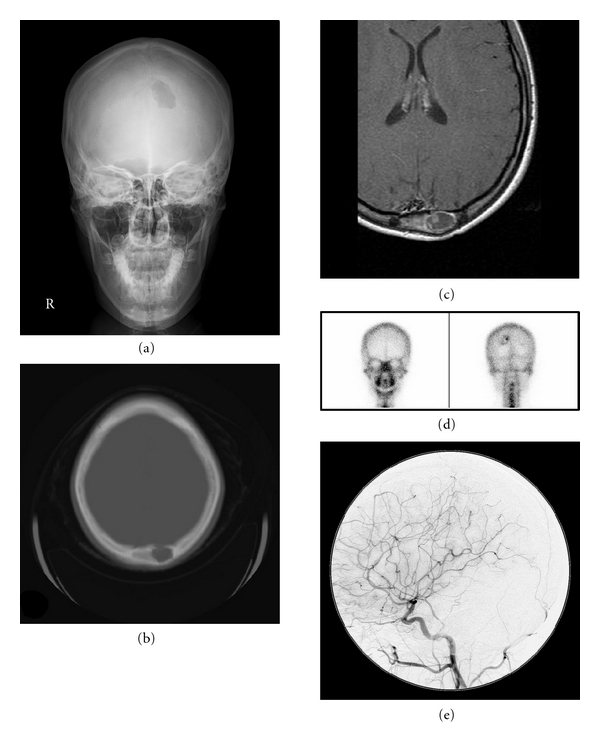
AP view of skull xp (a) showing the bony defect of the left occipital lesion. Bone image CT at the level of the subcutaneous mass (b) demonstrating the bony defect of the left occipital bone without a change in the intraaxial lesion. The skull surface of the defective part was swollen. Axial T1-weighted MRI with contrast medium (c) showing a cystic change corresponding to the bony defect and partial enhancement around the cystic change. ^99m^Tc-HMDP bone scintigraphy (d) showing accumulation of tracer in the part of the bone cyst corresponding to the strongly enhanced lesion found on MRI. Head DSA study (e, lateral view) demonstrating no abnormal vessels, including tumor feeder or aneurysmal changes.

A 14-year-old female had a bony defect on her head that was discovered by X-ray Photograph (xp) and computed tomography (CT) imaging, which were performed to investigate a head injury. The lesion was associated with scalp sutures she had received for a head injury she sustained at the age of 6. Immediately below the injury, a gradually enlarging bone-like tumor mass was observed. Upon admission, a subcutaneous mass with the same hardness as bone was found, which was palpable on the left occipital bone, and head xp showed a corresponding bone transmission image at the same lesion (Figure 1(a)). CT demonstrated a corresponding image of a bony defect and swelling of the outer table of the skull (Figure 1(b)). MRI revealed a corresponding bony defect and swelling at the subcutaneous tumor mass with cystic changes in the cavity of the bony defect, and a contrast study showed a partial enhancement around the cystic change (Figure 1(c)). ^99m^Tc-HMDP bone scintigraphy demonstrated strong accumulation at a part of the cystic lesion (Figure 1(d)), and head DSA study revealed no tumor stain or feeder (Figure 1(e)).

Based on clinical symptoms, history, and image observations, the differential diagnoses included aneurysmal bone cyst, simple bone cyst, eosinophilic granuloma with cystic change, growing skull fracture, and cystic FD. Therefore, a biopsy and tumorectomy under general anesthesia were performed to diagnose and radically cure the patient.

**Figure 2:**
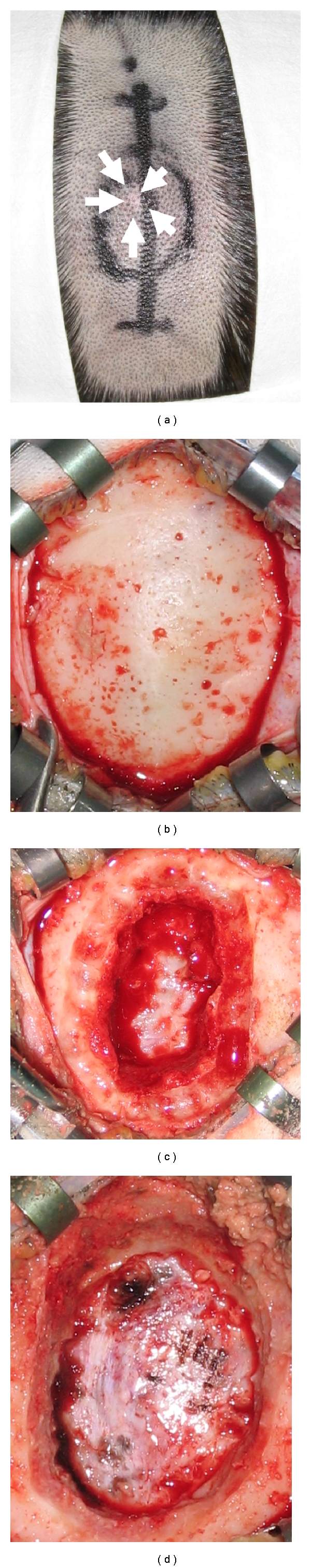
Intraoperative photographs (a–d). Photograph of the skin immediately above the skull mass (a) showing the cutaneous scar (arrowheads) from the past injury. The skull surface just below the skin scar was smooth but swollen (b). Photograph obtained after piercing the cyst (c) demonstrating bloody cyst fluid and the inner surface of the cyst wall with no obvious tumorous change. Intact dura mater was observed after total removal of the skull mass (d).

*Intraoperative Observations.* A cutaneous scar due to past injury was observed at the surface of skin immediately above the skull mass (Figure 2(a), arrowheads). The outer table of the skull beneath the skin of the scar was smooth without fracture but swollen (Figure 2(b)). After removing the outer table of the skull, the cystic cavity and bloody liquid component were observed. After aspiration of the liquid component, only bony tissue was grossly observed inside the cavity without macroscopic tumorous change, and the inner table of the skull was maintained with mild thinning (s). Finally, the cystic bony mass was completely removed, securing a margin of 5 mm around the mass. The dura mater and brain tissue just below the bony cyst was intact (Figure 2(d)). The defective part of bone was reconstructed with calcium phosphate paste. 

**Figure 3:**
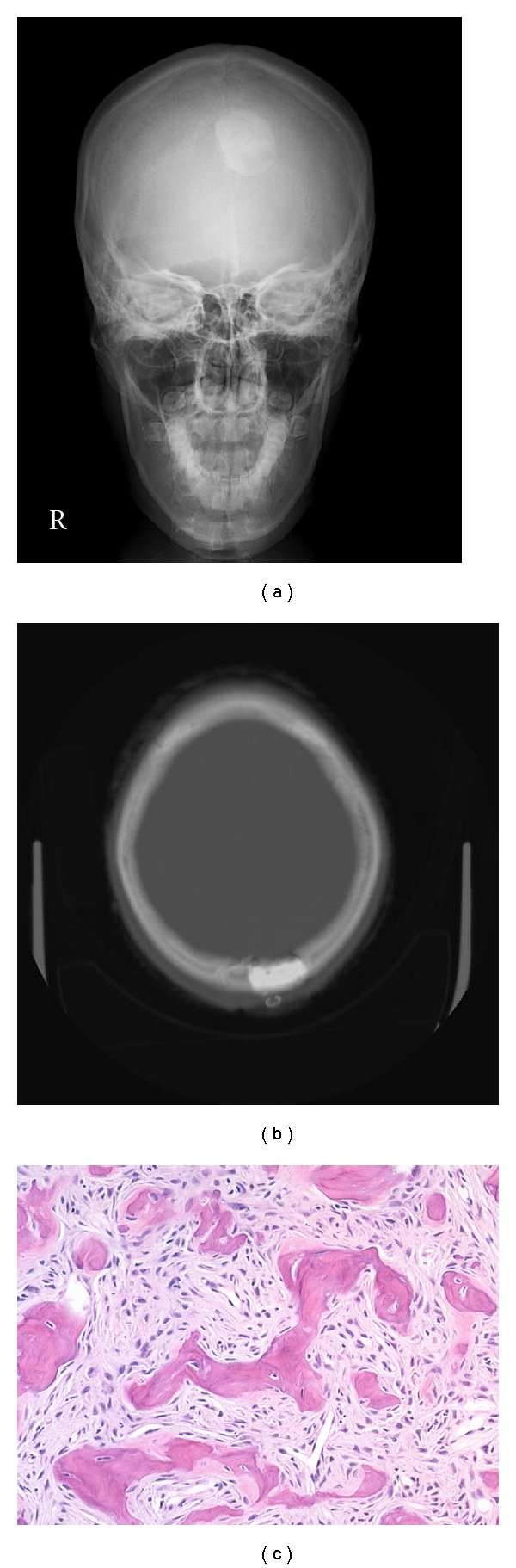
Postoperative AP view of skull xp (a) and bone image with head CT (b) showing complete resection of the tumor and excellent remodeling of the skull deficit using calcium phosphate paste. Microscopic view of the intraoperative biopsied specimen (c, H&E stain, ×200). The lesion was composed of irregularly shaped trabeculae of woven bone with calcification in a background of fibrous tissue. There were no osteoblasts on the surface of the trabeculae. These histological findings were indicative of fibrous dysplasia.

There were no significant postoperative complications. Postoperative xp and CT showed that the tumor mass had been completely removed, and cranioplasty of the bone deficit after tumor removal was successful (Figures 3(a) and 3(b)). Results of a pathological examination by H&E staining demonstrated an FD tissue image in a region where strong technetium accumulation was observed (Figure 3(c)).

## 3. Discussion

It can be difficult to discriminate FD, especially cystic types, from other progressive bone tumor diseases [4, 5] because radiographic findings can vary depending on the histological subtypes and components of the cyst. On the basis of the position of the lesion and radiographic findings, we considered a solitary bone cyst, aneurismal bone cyst, telangiectatic osteosarcoma, growing skull fracture, and eosinophilic granuloma with cystic change for diagnosis of our case [4, 5]. However, further differential diagnosis based on images alone was difficult, so a surgical diagnosis was required. 

One factor that made preoperative diagnosis of this case difficult was the history of external injury as well as radiographic findings. In our case, the progressive head tumor mass developed immediately after the head injury that was sustained at a young age, and the relationship between tumor onset and external injury was unclear. In some cases, skull tumor-like lesions reportedly started growing after external injuries [6, 7], but there have been no reported cases of FD triggered by head injury. Therefore, in the preoperative differential diagnosis with the images shown above, eosinophilic granuloma with cystic change was strongly suspected, rather than FD. Therefore, in this case, diagnosis based on the images and clinical course was difficult. The detailed mechanism underlying head trauma and pathogenesis of FD is still unknown, and it is possible that the relationship between FD and the head trauma is merely coincidental. However, a relationship between FD pathogenesis and increased expression of the *c-fos* gene has been reported [8], and upregulation of the *c-fos* gene in bone in relation with trauma has also been previously described [9]. Therefore, there is also a possibility of induction of FD by trauma via a *c-fos* gene-mediated mechanism.

From the histopathological aspect, tumor activity was observed only where bone scintigraphy indicated strong accumulation. On the other hand, there was minimal correlation between MRI findings and neoplastic change. Therefore, it is important to completely remove a site that can be radiographically visualized by contract MRI but shows strong accumulation with bone scintigraphy. This result is in agreement with previous reports that a site imaged by MRI does not always correlate with clinical and histological activity and that bone scintigraphy reflects the true extent of FD [10]. 

To our knowledge, this is the first report discussing the relationship between cranial FD and head trauma. It was difficult to make a diagnosis based on the clinical course and imaging. Therefore, biopsy and surgical resection were required. However, there is no need to perform a risky extirpative surgery for FD unless there is an active lesion or a profound cosmetic problem. If a preoperative diagnosis is difficult, as in this case, surgery will probably be required to make a definite diagnosis, even for lesions with lower activity.
